# Gift of nature: non-human-derived exosomes showing application prospects in periodontitis treatment

**DOI:** 10.3389/fimmu.2026.1814654

**Published:** 2026-09-02

**Authors:** Hongbo Fei, Zhuoran Wang, Yujiao Li, Hongchen Sun, Xiuping Meng, Hongbing Lin

**Affiliations:** 1 Hospital of Stomatology, Jilin University, Changchun, China; 2 Jilin Provincial Key Laboratory of Oral and Craniofacial Diseases & Tissue Reconstruction, Jilin University, Changchun, China; 3 Department of Periodontology, College of Stomatology, Xi’an Jiaotong University, Xi’an, China

**Keywords:** angiogenesis, immunomodulation, non-human-derived exosomes, periodontal inflammation, periodontal regeneration

## Abstract

Periodontitis, a chronic inflammatory disorder, results in tooth loss and adversely affects oral and systemic health. Current therapies fail to effectively regenerate bone loss caused by inflammation. Human stem cell-derived exosomes have emerged as important mediators of tissue regeneration and inflammation modulation in periodontitis, yet their clinical use faces ethical and technical challenges. Non-human-derived exosomes present a viable alternative, leveraging their ability to enhance tissue repair, reduce inflammation, and stimulate regeneration. This review synthesizes recent advances (2020–2025) in non-human-derived exosome applications, emphasizing their roles in immunomodulation, angiogenesis, and periodontal regeneration, while evaluating clinical translation prospects.

## Graphical Abstract

**Figure:**
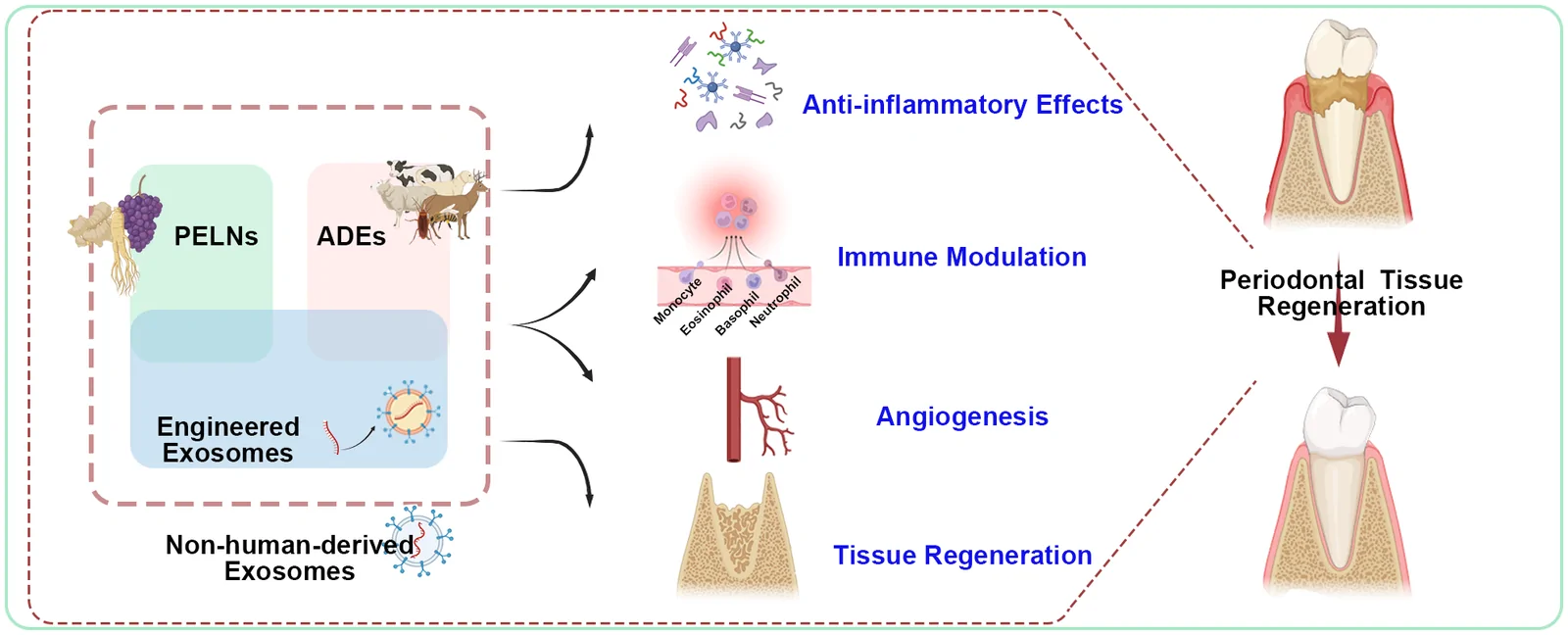
Therapeutic effects of non-human-derived exosomes and their potential as a therapeutic approach for periodontitis. Non-human-derived exosomes (PELNs, ADEs, and their engineered derivatives) have demonstrated anti-inflammatory, immune-modulatory, tissue-regenerative, and angiogenic effects that play a key role in periodontitis treatment.

## 1 Introduction

Periodontitis, marked by progressive destruction of periodontal structures, is a global health burden linked to systemic conditions like diabetes and cardiovascular diseases (1, 2). The Fourth National Oral Health Survey shows that about 62.3% of adults aged 35 and above in China suffer from varying degrees of periodontitis, and the severity is positively correlated with age (3). At present, non-surgical and surgical methods are commonly used, combined with various biomaterials and biological media to reconstruct damaged periodontal tissue, thereby improving the prognosis and quality of life of patients. However, the effects are limited (4, 5).

Recent studies of cellular communication have highlighted the role of exosomes in mediating tissue regeneration and modulating inflammatory responses associated with periodontitis. Exosomes are nano-sized vesicles secreted from various cells that facilitate intercellular communication and influence the behavior of recipient cells (6). Their unique characteristics, such as low immunogenicity, biocompatibility, and delivery capacity, make them promising candidates for therapeutic applications (7). Exosomes derived from mesenchymal stem cells (MSCs) have been shown to enhance periodontal regeneration (8). Exosome-based strategies have highlighted the potential to promote periodontal regeneration through regulation of immune cells and stem cells (6). Although exosomes derived from humans have shown promise in periodontal treatment, their clinical application is constrained by several major issues, including (1) the difficulty of obtaining human MSCs and associated ethical issues; (2) low production yields; and (3) the time-consuming and laborious production process (9, 10). Exosomes derived from non-humans offer an intriguing substitute. They come from a wide range of sources and have similar or even higher biocompatibility. Notably, their therapeutic value lies in their capacity to improve tissue healing, diminish inflammation, and promote tissue regeneration, which positions them as viable choices for novel treatments of periodontitis (9).

However, a vast majority of current systematic reviews remain focused on human-derived exosomes, while systematic reviews centered on non-human-derived exosomes remain scarce. To address this research gap, the present review focuses exclusively on non-human-derived exosomes and systematically consolidates the latest research advancements from the last 5 years, covering exosomes from animal, plant, and engineered sources. This work comprehensively integrates their underlying mechanisms in regulating inflammation, immune responses, angiogenesis, and periodontal tissue regeneration, while critically discussing the limitations of cross-disease extrapolation and the core challenges hindering clinical translation. By highlighting the unique therapeutic properties of non-human-derived exosomes in periodontitis therapy, this review aims to provide a comprehensive and innovative perspective to enrich the current body of relevant literature.

## 2 Sources and application of non-human-derived exosomes

Accumulating evidence demonstrates that plant-derived exosome-like nanovesicles share highly similar biogenesis pathways with mammalian-derived exosomes, mainly through the multivesicular bodies (MVBs) pathway (11). To accurately reflect the specific nomenclature of the original cited studies, we utilized terms such as ‘exosomes’, ‘exosome-like nanoparticles’, or ‘nanovesicles’ throughout this manuscript, which collectively fall under the broader, standardized umbrella of non-human-derived exosomes. Similar to exosomes derived from humans, non-human-derived exosomes are classified based on their origin, including animal, plant, and engineered sources, and also play a crucial role in intercellular communication.

### 2.1 Animal-derived exosomes

Human-derived exosomes have been extensively studied for their therapeutic potential. For instance, exosomes derived from MSCs show promise in numerous medical applications, including neuroprotection and tissue repair (12). However, ethical issues and difficulties in isolation constrain the clinical application of exosomes derived from human MSCs. Plantz et al. demonstrated the abundance of exosomes in bovine milk and their role in treating diseases (13). Therefore, animal-derived exosomes (ADEs) from various sources were subsequently identified and shown to promote treatments in both humans and animals (14, 15).

### 2.2 Plant-derived exosome-like nanoparticles

Plant-derived exosome-like nanoparticles (PELNs; 50–500 nm) are enriched with lipids like phosphatidic acid, facilitating cross-species communication (16). Derived from fruits, vegetables, and herbs, PELNs are cost-effective and scalable and exhibit low immunogenicity (11). In addition, their potential for large-scale production gives PELNs great potential for application (9).

### 2.3 Engineered exosomes

Surface modification and cargo loading [e.g., drugs and microRNAs (miRNAs)] enhance exosome targeting and therapeutic efficacy (9). Techniques like membrane hybridization and 3D-printed scaffolds optimize delivery for periodontal applications (17). For instance, engineered exosomes represent a novel approach in the field of drug delivery, where exosomes are modified to enhance their therapeutic efficacy (18).

Non-human-derived exosomes from different sources exhibit distinct therapeutic merits and limitations. ADEs, especially those isolated from milk, feature high yields, favorable cost efficiency, and superior biocompatibility, whereas they are associated with potential risks, including batch-to-batch inconsistency and cross-species viral transmission (19). PELNs support large-scale production and possess low intrinsic immunogenicity with proven dietary safety (9). Nevertheless, high-purity extraction and the standardization of cross-kingdom communication mechanisms remain major challenges (20). Engineered exosomes enable precise targeting and optimized cargo delivery; still, key challenges in engineered exosome translational research center on the lack of standardized protocols for isolation and clinical quantification, and the optimization of exosome source selection to match specific functional requirements (21).

## 3 Extraction and purification techniques

Ultracentrifugation, size-exclusion chromatography, and commercial isolation kits are the most common methods for exosome extraction. Ultracentrifugation is the traditional gold standard, providing high yields but requiring significant time and expertise (22). However, ultracentrifugation can lead to co-isolation of proteins and other contaminants, affecting the purity of the final exosome preparation (23). Size-exclusion chromatography offers a gentler approach that not only preserves the integrity of exosomes but also effectively separates them from contaminants. Furthermore, size-exclusion chromatography tends to yield purer exosome fractions but may result in lower overall yields (24). Commercial kits offer convenience and user-friendliness but are costly and may not always provide the highest purity.

## 4 The potential role of non-human-derived exosomes in periodontitis therapy

**Figure 1:**
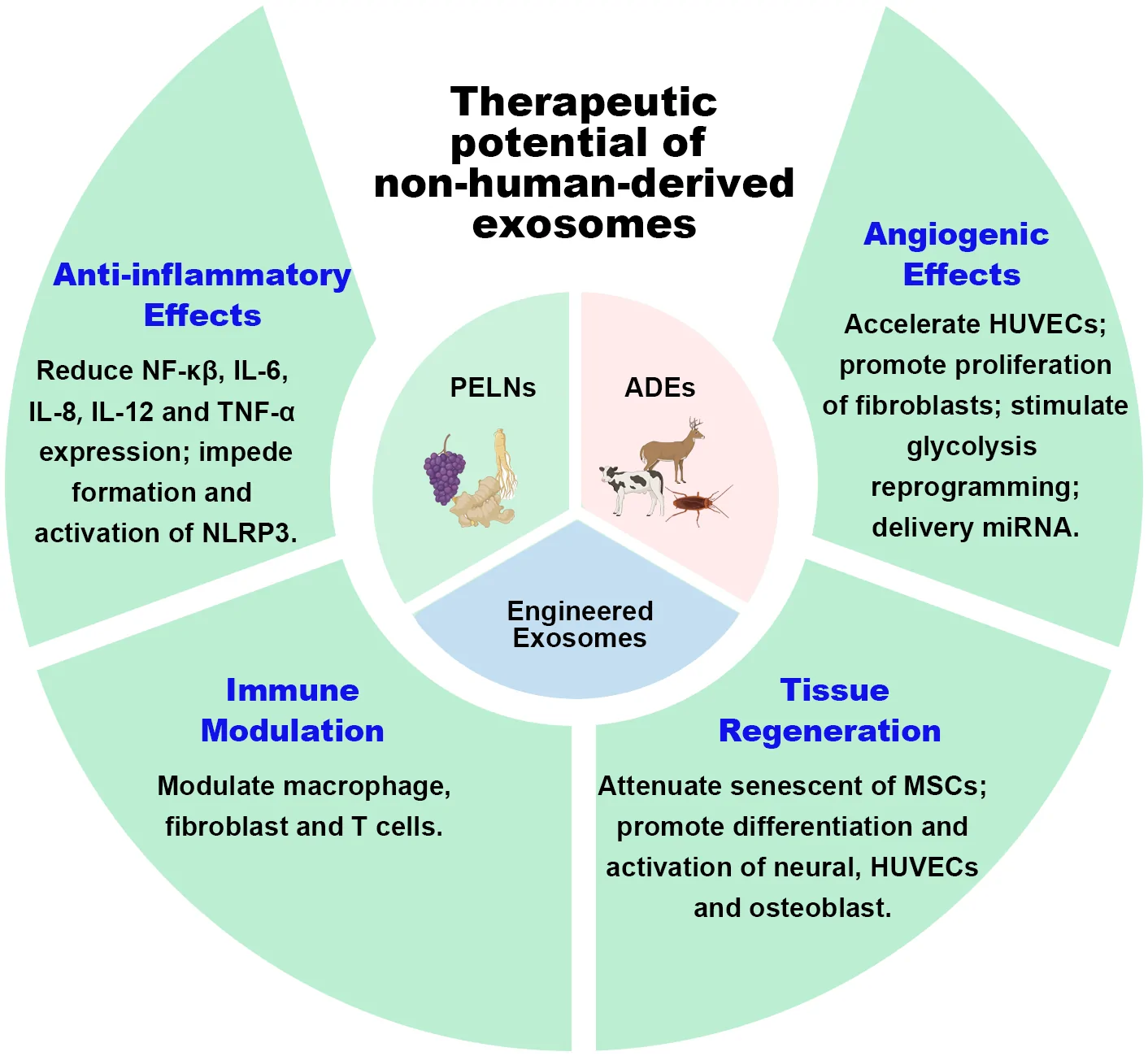
Biological activities of non-human-derived exosomes that may facilitate periodontal regeneration. Created with Biorender.com.

Periodontal tissue has a complex composition, which presents challenges for tissue engineering strategies (25). Anti-inflammatory activity, immune regulation, tissue regeneration, and angiogenesis are key components of periodontal tissue engineering. Literature reports reveal various biological activities of extracellular vesicles from non-human sources. These biological activities can promote periodontal tissue regeneration (**Figure 1**).

### 4.1 Anti-inflammatory effects

Traditional treatment methods for periodontitis, which focus on eliminating pathogenic microorganisms and using adjunctive pharmacotherapy, may result in resistance and increased rates of recurrence (26). In recent decades, people have attached increasing importance to using natural foods to treat human diseases. Xie et al. demonstrated that ginseng-derived exosome-like nanoparticles (GELNs) mainly alleviate inflammatory responses by suppressing the nuclear factor kappa B (NF-κB) signaling pathway (27). In addition, Yin et al. investigated the anti-inflammatory effects of GELNs. The results demonstrated that GELNs may counteract inflammation induced by lipopolysaccharide through miRNAs enriched in GELNs (28).

Animal-derived exosomes are a natural nutrient. Lu et al. showed that sheep milk-derived exosomes (sheep MDEs) may reduce interleukin-6 (IL-6) and interleukin-12 (IL-12) production by suppressing the toll-like receptor 4 (TLR4)/TRAF1-IκBα-p65 pathway through miRNAs (14). Chen et al. reported that vesicle-like nanoparticles in honey (H-VLNs) may impede the formation and activation of the pyrin domain-containing 3 (NLRP3) inflammasome through miR-4057 (29).

Luteolin (Lu) has notable bioactive properties, but its effects are hindered by water solubility and bioavailability. To overcome these disadvantages, Jiang et al. encapsulated Lu in sesame leaf-derived exosome-like nanovesicles (Exo@Lu) and demonstrated that Exo@Lu may improve the reduction in pro-inflammatory cytokines compared with free Lu (30). Detailed information on the anti-inflammatory effects of non-human-derived exosomes is listed in **Table 1**.

**Table 1:** Summary of studies on the anti-inflammatory effects of non-human-derived exosomes over the last 5 years.

Name	Source	NTA (nm)	Anti-inflammatory effects	Ref.
sheep MDEs	Sheep milk	87.95 ± 19.26	To reduce IL-6 and IL-12 production by suppressing the TLR4/TRAF1-IκBα-p65 pathway through miRNAs.	(14)
GELNs	Ginger	~161.2	To regulate oxidative stress and inflammatory reactions by inhibiting the NF-κB pathway.	(27)
GELNs	Ginger	156 ± 36	To downregulate inflammation through miRNAs.	(28)
H-VLNs	Honey	120–180	To impede the formation and activation of the NLRP3 inflammasome through miR-4057.	(29)
Exo@Lu	Lycium barbarum L.	151.45 ± 3.86	To reduce pro-inflammatory cytokine expression compared with free Lu.	(30)

### 4.2 Immune modulation

**Figure 2:**
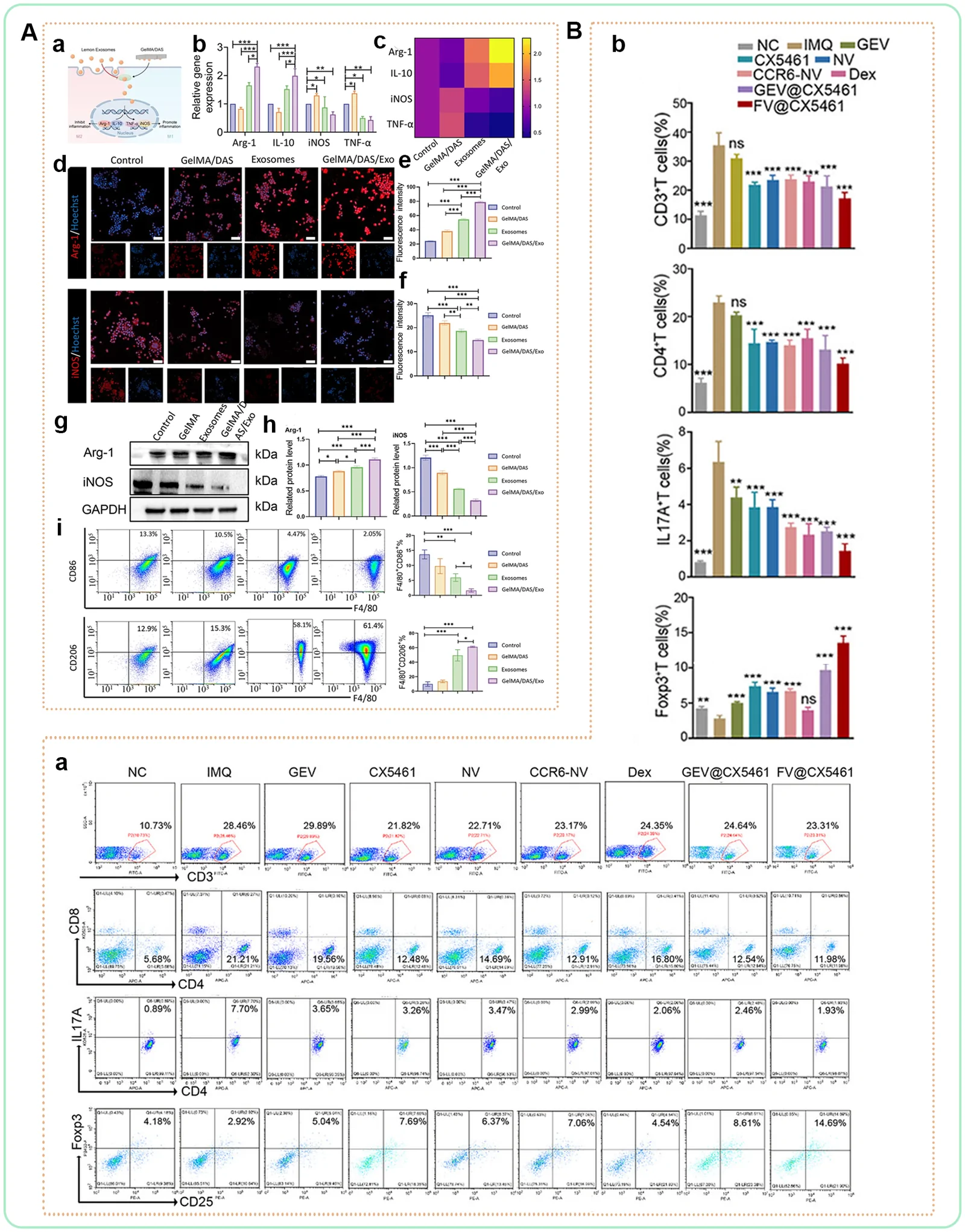
The immune-modulatory effects of non-human-derived exosomes. **(A)** The effect of non-human-derived exosomes on macrophage modulation. **(a)** Schematic diagram of GelMA/DAS/Exo modulation of macrophage polarization; **(b, c)**. Gene expression of cytokines; **(d–f)**. The fluorescence intensity of Arg-1 and iNOS. **(g, h)**. Protein expression of Arg-1 and iNOS; **(i)** The proportions of M1 and M2 macrophages. Reproduced with permission (35). **(B)** The effect of non-human-derived exosomes on T-cell modulation. **(a, b)**. Flow cytometric analysis of T cells in spleens from psoriasis. Reproduced with permission (39) *p<0.05; **p<0.01; ***p<0.001.

The immunomodulatory properties of non-human-derived exosomes extend beyond mere anti-inflammatory effects. These exosomes can influence immune cell behavior and enhance the overall immune response to periodontal pathogens (**Figure 2**).

The role of non-human-derived exosomes in modulating macrophages has been studied most extensively. First, non-human-derived exosomes may inhibit macrophage infiltration or recruitment. Zhang et al. demonstrated that exosomes derived from deer antler stem cells (AnSC-Exos) may inhibit circulating macrophage recruitment by inhibiting C-C motif chemokine ligand 7 (CCL7) expression in fibroblasts (31). Zhao et al. showed that garlic-derived exosome-like nanovesicles (GaELNVs) may ameliorate inflammatory eruptions by downregulating pro-inflammatory cytokine expression in the serum and hinder macrophage infiltration by inhibiting C-C motif chemokine receptor 2 (CCR2)/C-C motif chemokine receptor 5 (CCR5) signaling (32). Kim et al. showed that nanosized ginseng-derived exosome-like nanoparticles (GENs) exhibited strong efficacy in recruiting M1 macrophages (33). Second, non-human-derived exosomes play an important role in regulating macrophage polarization. Ou et al. showed that *Catharanthus roseus* (L.) Don leaf-derived exosome-like nanovesicles (CLDENs) may target immune organs, promote macrophage polarization, and promote lymphocyte proliferation *in vitro* (34). Lemon exosomes may regulate macrophage polarization, although limitations in drug delivery and penetration depth need to be addressed. Jin et al. loaded lemon exosomes into gelatin methacryloyl and dialdehyde starch to produce a GelMA-DAS-Lemon Exosomes hydrogel (GelMA/DAS/Exo hydrogel) and effectively promote the sustained release of exosomes (35). RAW264.7 macrophages were co-cultured with the GelMA/DAS/Exo hydrogel (**Figure 2(A)a)**. After 24 hours, expression of the M1-associated markers inducible nitric oxide synthase (iNOS) and tumor necrosis factor-alpha (TNF-α) was downregulated in both the Lemon Exosomes and GelMA/DAS/Exo groups, whereas the M2-associated markers arginase-1 (Arg-1) and interleukin-10 (IL-10) were upregulated (**Figures 2(A)b, c**). To recapitulate the inflammatory conditions characteristic of diabetic wounds, RAW264.7 cells were pre-stimulated with lipopolysaccharide (LPS) before hydrogel treatment. Immunofluorescence and Western blot analyses showed that exposure to either lemon exosomes or the GelMA/DAS/Exo hydrogel reduced the proportion of iNOS-positive M1 macrophages and increased that of Arg-1-positive M2 macrophages (**Figures 2(A)d–h**). These findings were further corroborated by flow cytometry, which revealed a decrease in CD86^+^ cells from 13.3% to 2.05% and an increase in CD206^+^ cells from 12.9% to 61.4% (**Figure 2(A)i**. Third, non-human-derived exosomes may regulate molecule expression. Martínez Fajardo et al. showed that exosomes derived from saffron tepals selectively stimulate macrophages and may increase CD80 and CD86 expression (36). Astaxanthin (AST) has excellent anti-inflammatory activity, but its limited biocompatibility restricts its applications. To overcome the disadvantages of AST, Cui et al. constructed hyaluronic acid-modified milk exosome-based astaxanthin (HA-mExo). The delivery system may accumulate in macrophages and significantly inhibit the expression of pro-inflammatory factors (37).

In addition, non-human-derived exosomes have also been reported to modulate T cells. Zhu et al. showed that *Portulaca oleracea* L.-derived exosome-like nanoparticles (PELNs) may activate the aryl hydrocarbon receptor on the surface of CD4+ T cells and reprogram T cells into double-positive CD4^+^CD8^+^ T cells (38). To improve efficacy in reaching the target tissue, Huang et al. encapsulated CX5461 in grapefruit-derived exosome-like nanovesicles (GEVs), which were then fused with engineered gingiva-derived MSCs to produce FV@CX5461. FV@CX5461 may reshape the unbalanced immune microenvironment by reducing inflammatory factor expression, downregulating Th17 activation, and promoting Treg infiltration (39). Flow cytometry confirmed the therapeutic effect of FV@CX5461. Compared with the other groups, FV@CX5461 markedly reduced both the absolute count of CD3^+^ T cells and the percentage of CD4^+^ T cells. It also decreased the proportion of splenic Th17 cells while increasing the frequency of CD4^+^CD25^+^Foxp3^+^ regulatory T cells (Tregs) (**Figures 2(B)a, b**). Detailed information on the immune-modulatory effects of non-human-derived exosomes is listed in **Table 2**.

**Table 2:** Summary of studies showing the immune-modulatory effects of non-human-derived exosomes over the last 5 years.

Name	Source	NTA (nm)	Immune modulation	Ref.
AnSC-Exos	deer antler	120	To inhibit macrophage recruitment by inhibiting CCL7 expression in fibroblasts.	(31)
GaELNVs	Garlic	43.82–396.1	To hinder macrophage infiltration by inhibiting CCR2/CCR5 signaling	(32)
GENs	Ginseng	151.6	To recruit M1 macrophages.	(33)
CLDENs	Catharanthus roseus	75.51 ± 10.19	To enhance macrophage polarization and lymphocyte proliferation.	(34)
GelMA/DAS/Exo hydrogel	Lemon	85–515	To regulate the polarization reprogramming of macrophages.	(35)
Exosomes derived from Saffron tepals	Saffron tepals	151.5 ± 79.6	To increase the expression of surface molecules on macrophages.	(36)
HA-mExo	Milk	100	To accumulate in macrophages and inhibit the expression of inflammatory factors.	(37)
PELNs	Portulaca oleracea L	~ 160	To reprogram CD4+ T cells into CD4^+^CD8^+^ T cells.	(38)
FV@CX5461	Grapefruit	163.4	To downregulate Th17 activation and promote Treg infiltration.	(39)

### 4.3 Tissue regeneration

**Figure 3:**
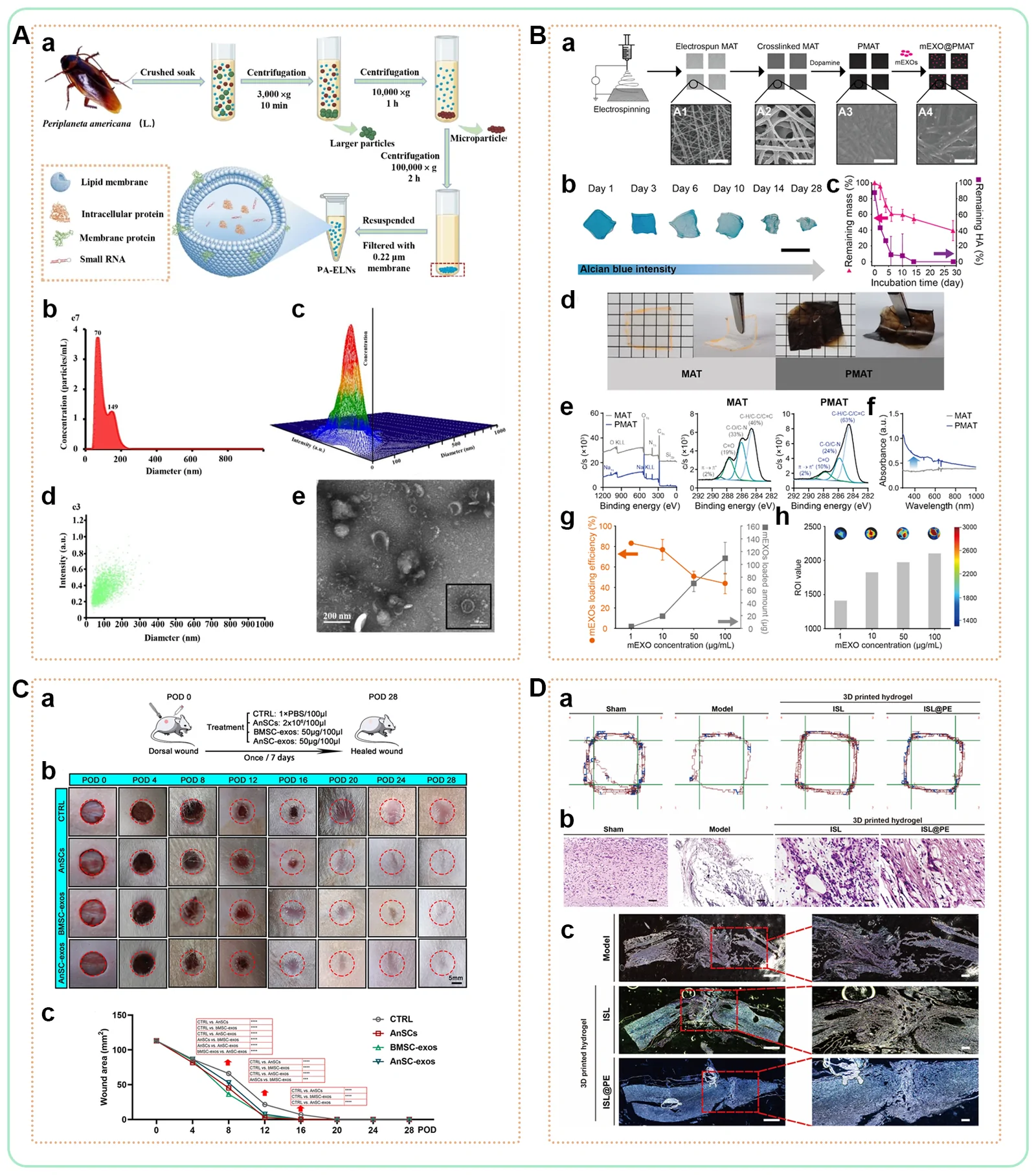
Tissue-regenerative effects of non-human-derived exosomes. **(A)** Isolation of PA-ELNs. **(a)** Isolation and purification of PA-ELNs; **(b–e)**. Characterization of PA-ELNs. Reproduced with permission (40). **(B)** Characterization of mEXO@PMAT (engineered exosome). **(a)** Preparation of mEXO@PMAT. **(b–f)**. Characterization of mEXO@PMAT; **(g)** mEXO loading on PMAT; **(h)** Concentrations of mEXO@PMAT. Reproduced with permission (41). **(C)** Effects of AnSC-exos on wound healing. **(a)** Experimental design; **(b, c)**. Photographs and evaluation of wound healing. Reproduced with permission (42). **(D)** The effect of ISL@PE in treating spinal cord injury. **(a)** Open field test; **(b)** Nissl staining; **(c)** HE staining. Scale bar = 50 μm. Reproduced with permission (46).

Non-human-derived exosomes, rich in growth factors and bioactive molecules, facilitate cellular processes essential for tissue regeneration. Recently, numerous studies on non-human-derived exosomes have demonstrated tissue-regenerative effects (**Figure 3**).

To explore the remarkable regenerative capacity of *Periplaneta americana* L. (PA), Liao et al. isolated PA-derived exosome-like nanoparticles (PA-ELNs) and demonstrated that they may promote the proliferation and recruitment of human umbilical vein endothelial cells (HUVECs) and RAW 264.7 cells. The remarkable effect on accelerating wound healing may rely on miRNAs associated with some wound-healing signaling pathways (40). PA-ELNs were successfully isolated from the medicinal insect PA via differential velocity centrifugation and characterized (**Figures 3(A)a–e**). To our knowledge, this is the first report of exosome-like nanoparticle isolation from this medicinal insect, with physicochemical properties comparable to those of exosomes from other natural sources. In addition, to enhance the therapeutic effect on wound healing, Bui et al. immobilized pasteurized mEXOs onto a polydopamine (PDA)-coated hyaluronic acid (HA)-based electrospun nanofibrous matrix to produce a wound-healing biomaterial named mEXO@PMAT, which enabled the gradual release of mEXOs without a burst effect. The results demonstrated that the sustained release of mEXOs may significantly enhance cell proliferation and promote wound closure (41). The characterization results of MAT are presented in **Figures 3(B)a–c**, including surface morphology before and after crosslinking, mass erosion, and residual HA weight. Following PDA adhesion and polymerization, the originally transparent MAT turned dark brown (PMAT) due to visible-light absorption by PDA (**Figures 3(B)d, f**), and X-ray photoelectron spectroscopy (XPS) analysis confirmed the elemental composition of the PDA layer (**Figure 3(B)e**). fluorescein isothiocyanate (FITC)-labeled mEXOs revealed that the extent of mEXO immobilization on PMAT increased progressively with increasing mEXO concentration (**Figure 3(B)g**). This was further supported by *in vivo* imaging system (IVIS) analysis, which showed a concentration-dependent increase in fluorescence signal, indicating successful immobilization (**Figure 3(B)h**). Velvet deer antlers are the only known mammalian organs that can regenerate annually. Zhang et al. showed that AnSC-exos may effectively promote tissue regeneration by reducing fibroblast-to-myofibroblast transition (42). In a rat full-thickness wound model, AnSC-exos accelerated wound healing compared with phosphate-buffered saline (PBS) controls, with antler stem cells (AnSCs) and bone marrow mesenchymal stem cell-derived exosomes (bMSC-exos) serving as positive controls (**Figure 3(C)a**). Wound area measurements showed that on postoperative day 8 (POD8), closure efficacy ranked as bMSC-exos > AnSCs > AnSC-exos > CTRL, whereas on POD12, the order shifted to AnSCs > AnSC-exos > bMSC-exos > CTRL. By POD16, all treatment groups achieved near-complete closure, whereas CTRL wounds remained open until POD20 (**Figures 3(C)b, c**). Furthermore, Lei et al. showed that AnSC-exos may promote self-renewal and repress senescence-related inflammatory responses to attenuate senescent phenotypes in human MSCs and contribute to bone and cartilage regeneration (43). Xu et al. showed that ginseng-derived exosomes (G-Exos) may stimulate neural differentiation by transferring miRNAs to bone marrow mesenchymal stem cells (BMSCs) (44). Hwang et al. demonstrated that yam-derived exosome-like nanovesicles (YNVs) may promote osteoblast differentiation and mineralization by increasing osteogenic markers. Furthermore, the osteogenic activity of YNVs may result from regulation of the BMP-2/p-p38-dependent Runx2 pathway (45). Engineered exosomes have also been developed in the field of tissue regeneration in recent years. Wang et al. isolated plant-derived exosomes (PE) from *Lycium barbarum* L. and encapsulated the isoliquiritigenin (ISL)-loaded PE (ISL@PE) within a 3D-printed bionic scaffold. The results demonstrated that ISL@PE may improve neurological function and provide a potential route for insoluble drug delivery (46). The ISL@PE group demonstrated superior repair effects, as evidenced by open-field test results (**Figure 3(D)a**). Histological analysis showed spinal nerves in the 3D-printed hydrogel groups regenerated well (**Figure 3(D)b**). Notably, Nissl staining revealed a significant increase in the number of motor neurons in the caudal anterior horn of the spinal cord within the spinal cord injury (SCI) region in the ISL@PE group compared with the SCI model group (**Figure 3(D)c**). Detailed information on the tissue-regenerative effects of non-human-derived exosomes is listed in **Table 3**.

**Table 3:** Summary of studies showing the tissue-regenerative effects of non-human-derived exosomes over the last 5 years.

Name	Source	NTA (nm)	Tissue repair and regeneration	Ref.
PA-ELNs	Periplaneta americana L.	104.7	To promote HUVEC proliferation and migration and promote wound healing.	(40)
AnSC-exos	deer antler	120	To promote regenerative cutaneous wound healing by inhibiting fibroblast-to-myofibroblast transition (FMT).	(42)
AnSC-exos	deer antler	100	To attenuate senescent phenotypes in human MSCs and contribute to bone and cartilage regeneration.	(43)
G-Exos	Ginseng	144.1 ± 2.8	To stimulate neural differentiation by transferring miRNA to BMSCs.	(44)
YNVs	Yam	100	To promote osteoblast differentiation through the BMP-2/p-p38-dependent Runx2 pathway.	(45)
ISL@PE	Lycium barbarum L.	155.1 ± 3.3	To promote neuronal differentiation.	(46)
mEXO@PMAT	milk	~82	To accelerate wound closure *in vivo* and enhance cell proliferation *in vitro*.	(41)

### 4.4 Angiogenic effects

Angiogenesis is a critical component of tissue regeneration, particularly in the periodontal environment. This angiogenic effect not only aids in the restoration of blood supply to the periodontal tissues but also contributes to the overall healing process by facilitating the delivery of immune cells and nutrients to the site of injury.

Meng et al. showed that antler mesenchymal stem cell-derived exosomes (AMSC-Exo) may accelerate HUVEC migration and angiogenesis. Notably, miR-21-5p/signal transducer and activator of transcription 3 (STAT3) pathway plays an important role in increased vascularization (15). Tan et al. showed that ginseng-derived exosomes (GExos) may reverse endothelial cell function in a high-glucose environment for angiogenesis by stimulating glycolytic angiogenesis through reprogramming, including upregulation of anaerobic glycolysis and downregulation of oxidative phosphorylation (47). As mentioned above, the GelMA/DAS/Exo hydrogel developed by Jin et al. not only overcomes limitations in the frequency and depth of drug delivery but also enhances fibroblast and vascular endothelial cell proliferation and recruitment to promote wound healing (35). In addition, Yan et al. coated miR-31-5p mimics with milk-derived exosomes and showed that the miRNA-exosomal formulation dramatically promoted angiogenesis and enhanced diabetic wound healing (48). Detailed information on the angiogenic effects of non-human-derived exosomes is listed in **Table 4**.

**Table 4:** Summary of studies on the angiogenic effects of non-human-derived exosomes over the last 5 years.

Name and	Source	NTA (nm)	Angiogenic effects	Ref.
AMSC-Exo	Deer antler mesenchymal stem cells	150	To accelerate HUVEC angiogenesis and stimulate angiogenesis through the miR-21-5p/STAT3 pathway	(15)
Lemon exosomes	Lemon	85–515	To promote fibroblast and vascular endothelial cell proliferation and migration.	(35)
GExos	Ginseng	117.7 ± 6.3	To stimulate glycolysis angiogenesis through reprogram.	(47)
miRNA-exosomal formulation	Milk	131.1	To serve as a delivery system for miR-31-5p and promote angiogenesis.	(48)

Despite encouraging results from various disease models, the direct translation of these findings to periodontal therapy remains constrained by the distinctive nature of the periodontal microenvironment. Future investigations should therefore validate these mechanistic insights in standardized *in vivo* models of periodontal defects.

## 5 Clinical trial progress

Recent clinical studies have focused on the efficacy of non-human-derived exosomes in promoting periodontal regeneration. For example, a randomized controlled trial evaluated the impact of locally delivered plant stem cell-derived exosomes on patients with stage III periodontitis. Results indicated significant improvements in clinical parameters, suggesting that these exosomes can effectively enhance periodontal healing (49).

While clinical trials specifically investigating non-human-derived exosomes for the treatment of periodontitis remain nearly absent, a comprehensive search across global databases, including the WHO International Clinical Trials Registry Platform (ICTRP), EU Clinical Trials Register (EU-CTR), ClinicalTrials.gov, and Chinese Clinical Trial Registry (ChiCTR) [keywords: “extracellular vesicles (EVs)” or “exosomes”], revealed seven registered clinical trials focused on non-human-derived EVs for various inflammatory conditions, as shown in **Table 5**. These trials highlight a pivotal shift: the therapeutic potential of non-human-derived EVs is transcending laboratory research and entering the phase of clinical validation. Although they target different sites, these trials provide essential cross-disciplinary evidence regarding the safety, dosage, and immunomodulatory efficacy of non-human-derived EVs. Such progress establishes a robust foundation for their future application in periodontal tissue regeneration and inflammatory control.

**Table 5:** Clinical trials of exosome treatment for inflammatory diseases over the last 5 years.

Source	Target condition/disease	Trial phase	Registration number	Current status
Hybrid exosomes for targeted delivery of CRISPR/Cas9 gene editing of MMP-13	cartilage defect	Outerbridge grade III/IV	ChiCTR2100041827	Prospective registration
Exosome-Delivered Baicalin	Chronic Rhinosinusitis	Diagnosis of chronic rhinosinusitis with nasal polyps (CRSwNP) (EPOS2020 criteria) with type 2 inflammation	ChiCTR2500103992	Prospective registration
3D extracellular vesicles	Knee osteoarthritis	Kellgren–Lawrence grade 2–3 by x-ray	ChiCTR2500115601	Prospective registration
Rothia mucilaginosa-derived membrane vesicles	actinic cheilitis	Rubem grade II or above	ChiCTR2500100015	Retrospective registration
Grape exosomes	Oral Mucositis Associated With Chemoradiation Treatment of Head and Neck Cancer	Eastern Cooperative Oncology Group (ECOG) performance status 0, 1, or 2 (Karnofsky > 60%)	NCT01668849	Completed
Allogeneic MSC-derived exosomes	Autoinflammatory and Post-infectious Neuroinflammatory Syndromes	/	NCT07145502	Active
Plant Exosomes with/without Curcumin	Inflammatory Bowel Disease	moderate disease activity	NCT04879810	Completed

## 6 Summary and prospects

Despite the significant potential of non-human-derived exosomes as innovative regenerative strategies for periodontal therapy, several pivotal challenges must be systematically resolved to bridge the gap between preclinical promise and clinical translation.

First, the primary bottleneck lies in the lack of unified protocols for exosome isolation, characterization, and quality control (QC). To ensure reproducibility, detailed operational parameters during isolation, such as centrifugation speed, rotor type, duration, temperature, tube selection, sample volume, and brake settings, must be explicitly documented. Furthermore, given that non-specific assays [e.g., NTA and bicinchoninic acid (BCA)/Bradford] can misrepresent purity because of non-exosome contaminants, relying on a single analytical tool is insufficient. Comprehensive characterization is imperative, integrating (1) electron microscopy (EM) and NTA for morphological and single-particle verification; (2) Western blotting or ELISA to confirm specific biomarkers (e.g., CD9 and CD81 as positive markers and calnexin as a negative marker); and (3) total protein, lipid, and RNA content to ensure batch-to-batch consistency and therapeutic potency (50, 51).

Second, advancing exosome-based therapeutics to market approval requires strict compliance with international regulatory standards. Manufacturing processes must adhere to International Council for Harmonization (ICH) guidelines, specifically utilizing the Common Technical Document (CTD) format, and incorporate good manufacturing practice (GMP)-compliant, xeno-free raw materials. Additionally, robust downstream processing strategies must be established to optimize storage, formulation, and delivery routes, thereby preserving the physicochemical integrity and bioactivity of exosomes during transport and clinical administration (51).

Finally, evaluating the biosafety of non-human-derived exosomes remains paramount before human trials. Because conventional toxicological assays may fail to capture the complex biological interactions of extracellular vesicles, novel evaluation frameworks are needed. Future investigations must rigorously assess long-term immunogenicity, host immune responses, and dose-dependent toxicity while establishing precise dosing regimens to ensure safe and effective clinical outcomes (51).

## 7 Conclusions

Non-human-derived exosomes originate from diverse sources and exhibit favorable biocompatibility comparable to that of human MSC-derived exosomes. Their potent capacities for facilitating tissue repair, suppressing inflammation, and promoting regeneration render them promising bioactive candidates for novel periodontitis therapies. Nevertheless, these exosome platforms are still confined to preclinical investigation and cannot yet fully replace human cell-derived exosomes. To bridge this gap, organ-on-a-chip technologies are increasingly utilized to accurately simulate the human periodontal microenvironment and evaluate exosome performance *in vitro*. From a translational viewpoint, the development of non-human-derived exosome-based periodontal therapeutics is advancing from basic bench research toward standardized clinical application. Future breakthroughs in periodontal tissue engineering are anticipated to rely on hybrid systems that integrate the inherent biosafety and abundant availability of plant or animal-derived exosomes with engineered bioscaffolds tailored to the complex oral microenvironment. Successful clinical translation requires standardized source-specific characterization, reproducible manufacturing procedures, rigorous validation of periodontal-specific efficacy, optimized local delivery strategies, and comprehensive long-term safety evaluation. With advances in interdisciplinary research, non-human-derived exosomes are expected to evolve from alternative biomaterials into mainstream first-line candidates for precise and functional periodontal regeneration.
